# Thermodynamics of nanodisc formation mediated by styrene/maleic acid (2:1) copolymer

**DOI:** 10.1038/s41598-017-11616-z

**Published:** 2017-09-14

**Authors:** Anne Grethen, Abraham Olusegun Oluwole, Bartholomäus Danielczak, Carolyn Vargas, Sandro Keller

**Affiliations:** 1 Molecular Biophysics, University of Kaiserslautern, 67663 Kaiserslautern, Germany; 2 Department of Chemistry, University of Ibadan, 200284 Ibadan, Nigeria

## Abstract

Styrene/maleic acid copolymers (SMA) have recently attracted great interest for *in vitro* studies of membrane proteins, as they self-insert into and fragment biological membranes to form polymer-bounded nanodiscs that provide a native-like lipid-bilayer environment. SMA copolymers are available in different styrene/maleic acid ratios and chain lengths and, thus, possess different charge densities, hydrophobicities, and solubilisation properties. Here, we studied the equilibrium solubilisation properties of the most commonly used copolymer, SMA(2:1), by monitoring the formation of nanodiscs from phospholipid vesicles using ^31^P nuclear magnetic resonance spectroscopy, dynamic light scattering, and differential scanning calorimetry. Comparison of SMA(2:1) phase diagrams with those of SMA(3:1) and diisobutylene/maleic acid (DIBMA) revealed that, on a mass concentration scale, SMA(2:1) is the most efficient membrane solubiliser, despite its relatively mild effects on the thermotropic phase behaviour of solubilised lipids. In contrast with previous kinetic studies, our equilibrium experiments demonstrate that the solubilisation of phospholipid bilayers by SMA(2:1) is most efficient at moderately alkaline pH values. This pH dependence was also observed for the solubilisation of native *Escherichia coli* membranes, for which SMA(2:1) again turned out to be the most powerful solubiliser in terms of the total amounts of membrane proteins extracted.

## Introduction

Amphiphilic copolymers—in particular, styrene/maleic acid (SMA) copolymers—have gained considerable attention over the past few years because of their ability to solubilise biological membranes into SMA-bounded nanodiscs containing membrane proteins and lipids^1–3^. This approach is independent of conventional detergents and results in nanosized membrane mimics that retain the bilayer architecture of the parent membrane^4, 5^. Polymer-mediated solubilisation renders membrane proteins amenable to functional^6–8^ and biophysical^7, 9^ studies as well as structural analysis by nuclear magnetic resonance (NMR) spectroscopy^10, 11^. Furthermore, SMA-bounded nanodiscs have recently been used to transfer membrane proteins into lipidic cubic phases for structure determination by X-ray crystallography^12^.

SMA is a random copolymer that is commercially available in different average styrene/maleic acid ratios and chain lengths and, consequently, different charge densities, hydrophobicities, and solubilisation properties. The most hydrophilic variant SMA(1:1) and the most hydrophobic variant SMA(4:1), which have average styrene/maleic acid molar ratios of 1:1 and 4:1, respectively, are of limited use for solubilising lipid vesicles because of the narrow pH windows within which these copolymers are sufficiently soluble and hydrophobic. By contrast, SMA(2:1) and SMA(3:1) are capable of forming lipid-bilayer nanodiscs over a broader range of pH values and have become the two most popular amphiphilic copolymers used for this purpose. SMA(2:1) has been shown to be the most favourable solubiliser of three different membrane proteins^13^ and is emerging as the standard SMA variant for membrane-protein research using polymer-bounded nanodiscs^14^. While the structural properties^5^ and the self-association^15^ of SMA(2:1) as well as the kinetics of vesicle solubilisation mediated by this copolymer^15^ have been studied in great detail, only little is currently known about its solubilisation thermodynamics. One observation from kinetic experiments that remains particularly puzzling is that the solubilisation performance of SMA(2:1) is higher than that of SMA(3:1) but appears to decrease with increasing pH^15^, although a higher maleic acid content—as in SMA(2:1) compared with SMA(3:1)—and elevated pH should have similar effects on the charge density and the effective hydrophobicity of the copolymer.

Herein, we provide a thermodynamic benchmark for a more detailed understanding of the interactions of SMA(2:1) with lipid membranes and, specifically, of the roles of polymer, lipid, and solvent properties. To this end, we present the first account of the equilibrium solubilisation properties of SMA(2:1) against large unilamellar vesicles (LUVs) composed of either 1,2-dimyristoyl-*sn*-glycero-3-phosphocholine (DMPC) or 1-palmitoyl-2-oleoyl-*sn*-glycero-3-phosphocholine (POPC) as monitored by ^31^P NMR spectroscopy, dynamic light scattering (DLS), and differential scanning calorimetry (DSC). We rationalised the solubilisation equilibrium in terms of a pseudophase concept, constructed phase diagrams, and obtained vesicle-to-nanodisc transfer free energies that enable a thermodynamic comparison with more hydrophobic SMA(3:1)^16, 17^ and less hydrophobic diisobutylene/maleic acid (DIBMA)^18^ copolymers. We found that, on a *mass* concentration scale, both the onset and the completion of solubilisation of DMPC and POPC LUVs require less SMA(2:1) than SMA(3:1) or DIBMA, thus showing that SMA(2:1) is the most efficient solubiliser of lipid membranes. Importantly, SMA(2:1)-mediated lipid solubilisation was thermodynamically more efficient at pH 8.3 than at pH 7.4, even though the solubilisation process has been reported to slow down at alkaline pH^15^. Our lipid-bilayer studies under equilibrium conditions rather than kinetic control correlate with experiments performed on protein-containing biological membranes, as we found SMA(2:1) to furnish the largest amounts of membrane proteins extracted from native *Escherichia coli* membranes, again with an improvement in solubilisation yield at pH 8.3 as compared with pH 7.4.

## Theoretical background

### Pseudophases in lipid/surfactant mixtures

We have shown^16–18^ that the solubilisation of DMPC and POPC LUVs by SMA(3:1) or DIBMA can be rationalised in terms of a three-stage model^19, 20^ that considers lipid (L) and surfactant (S) molecules in bilayer (b) and micellar (m) phases as well as surfactant monomers in the aqueous (aq) phase. The concentrations of lipid and surfactant, *c*_L_ and *c*_S_, respectively, determine the presence and abundance of each of these phases. In a lipid/polymer mixture, where the polymer assumes the role of the surfactant, an increase in *c*_S_ at given *c*_L_ leads to a transition from the vesicular bilayer range to the coexistence range, within which polymer-saturated bilayer vesicles coexist with lipid-saturated nanodiscs. Upon a further increase in *c*_S_, the vesicles are completely solubilised and transformed into polymer-bounded nanodiscs. In this interpretation of the three-stage model, nanodiscs take the role of mixed micelles found in conventional lipid/surfactant mixtures^19, 20^. The first nanodiscs are formed at a threshold known as the saturation (SAT) boundary, while a second transition designated as the solubilisation (SOL) boundary marks the completion of nanodisc formation and the concomitant disappearance of the last vesicular structures.

Plotting the *c*_S_ values at the SAT and SOL boundaries against the corresponding lipid concentrations *c*_L_ gives rise to two straight lines described by:

1 $${c}_{{\rm{S}}}^{{\rm{SAT}}}={c}_{{\rm{S}}}^{{\rm{aq}},{\rm{o}}}+{R}_{{\rm{S}}}^{{\rm{b}},{\rm{SAT}}}{c}_{{\rm{L}}}$$

2 $${c}_{{\rm{S}}}^{{\rm{SOL}}}={c}_{{\rm{S}}}^{{\rm{aq}},{\rm{o}}}+{R}_{{\rm{S}}}^{{\rm{m}},{\rm{SOL}}}{c}_{{\rm{L}}}$$

The slopes ${R}_{{\rm{S}}}^{{\rm{b}},{\rm{SAT}}}$ and ${R}_{{\rm{S}}}^{{\rm{m}},{\rm{SOL}}}$ denote the polymer/lipid molar ratios in vesicular bilayers and nanodiscs at which the vesicles become saturated with polymer and at which solubilisation is complete, respectively. Ideally, both lines meet at a common ordinate intercept, ${c}_{{\rm{s}}}^{{\rm{aq}},{\rm{o}}}$, which corresponds to the concentration of “free” polymer in the aqueous phase within the coexistence range. In both our previous^16–18^ and present phase diagrams, the ordinate intercepts of the SAT and SOL boundaries are negligibly low, so that the concentration of “active” (i.e., solubilisation-competent) polymer in the aqueous phase can be taken as ${c}_{{\rm{S}}}^{{\rm{a}}{\rm{q}},{\rm{o}}}=0$.

The critical mole fractions of polymer in vesicular bilayers and nanodiscs, ${X}_{{\rm{S}}}^{{\rm{b}},{\rm{SAT}}}$ and $\,{X}_{{\rm{S}}}^{{\rm{m}},{\rm{SOL}}}$, respectively, amount to:

3 $${X}_{{\rm{S}}}^{{\rm{b}},\mathrm{SAT}\,}=\frac{{R}_{{\rm{S}}}^{{\rm{b}},{\rm{SAT}}}}{1+{R}_{{\rm{S}}}^{{\rm{b}},{\rm{SAT}}}}$$

4 $${X}_{{\rm{S}}}^{{\rm{m}},{\rm{SOL}}}=\frac{{R}_{{\rm{S}}}^{{\rm{m}},{\rm{SOL}}}}{1+{R}_{{\rm{S}}}^{{\rm{m}},{\rm{SOL}}}}$$

The partition coefficients quantifying the transfer of polymer and lipid from vesicles into nanodiscs, ${K}_{{\rm{S}}}^{{\rm{b}}\to {\rm{m}}}$ and ${K}_{{\rm{L}}}^{{\rm{b}}\to {\rm{m}}}$, are then given by:

5 $${K}_{{\rm{S}}}^{{\rm{b}}\to {\rm{m}}}\,\equiv \frac{{X}_{{\rm{S}}}^{{\rm{m}},{\rm{SOL}}}}{{X}_{{\rm{S}}}^{{\rm{b}},{\rm{SAT}}}}=\frac{{R}_{{\rm{S}}}^{{\rm{m}},{\rm{SOL}}}(1+{R}_{{\rm{S}}}^{{\rm{b}},{\rm{SAT}}})}{{R}_{{\rm{S}}}^{{\rm{b}},{\rm{SAT}}}(1+{R}_{{\rm{S}}}^{{\rm{m}},{\rm{SOL}}})} > 1$$

6 $${K}_{{\rm{L}}}^{{\rm{b}}\to {\rm{m}}}\equiv \frac{{X}_{{\rm{L}}}^{{\rm{m}},{\rm{SOL}}}}{{X}_{{\rm{L}}}^{{\rm{b}},{\rm{SAT}}}}=\frac{{\rm{1}}-{X}_{{\rm{S}}}^{{\rm{m}},{\rm{SOL}}}}{{\rm{1}}-{X}_{{\rm{S}}}^{{\rm{b}},{\rm{SAT}}}}=\frac{1+{R}_{{\rm{S}}}^{{\rm{b}},{\rm{SAT}}}}{1+{R}_{{\rm{S}}}^{{\rm{m}},{\rm{SOL}}}} < 1$$

From these partition coefficients, the corresponding standard molar Gibbs free-energy changes accompanying the transfer of the polymer and the lipid from vesicles into nanodiscs, ${\rm{\Delta }}{G}_{{\rm{S}}}^{{\rm{b}}\to {\rm{m}},{\rm{o}}}$ and ${\rm{\Delta }}{G}_{{\rm{L}}}^{{\rm{b}}\to {\rm{m}},{\rm{o}}}$, respectively, are obtained as:

7 $${\rm{\Delta }}{G}_{{\rm{S}}}^{{\rm{b}}\to {\rm{m}},{\rm{o}}}=-RT\,\mathrm{ln}\,{K}_{{\rm{S}}}^{{\rm{b}}\to {\rm{m}}} < 0$$

8 $${\rm{\Delta }}{G}_{{\rm{L}}}^{{\rm{b}}\to {\rm{m}},{\rm{o}}}=-RT\,\mathrm{ln}\,{K}_{{\rm{L}}}^{{\rm{b}}\to {\rm{m}}} > 0$$

### Derivation of phase boundaries from ^31^P NMR

According to the three-stage model, all phospholipid molecules and, thus, all phosphorus nuclei reside in bilayer membranes as long as the surfactant concentration is lower than or equal to ${c}_{{\rm{S}}}^{{\rm{S}}{\rm{A}}{\rm{T}}}$ according to equation 1. In solution-state NMR experiments, the signal arising from ^31^P nuclei in large, vesicular structures is broadened beyond detection. Thus, the area of the ^31^P NMR peak, *A*, is zero in the absence of solubilised phospholipid:

9 $$A({c}_{{\rm{S}}}\le {c}_{{\rm{S}}}^{{\rm{S}}{\rm{A}}{\rm{T}}})=0$$

Once the polymer concentration exceeds $\,{c}_{{\rm{S}}}^{{\rm{SOL}}}$ (equation 2), all phospholipid molecules are solubilised, and the area under the ^31^P NMR peak amounts to:

10 $$A({c}_{{\rm{S}}}^{{\rm{S}}{\rm{O}}{\rm{L}}}\le {c}_{{\rm{S}}})=f{c}_{{\rm{L}}}$$

where *f* is the proportionality factor between the concentration of solubilised lipids and the experimentally determined peak area. In general, *f* depends on the experimental conditions but is constant for a given NMR spectrometer operated using identical instrument settings and acquisition parameters. Within the coexistence range, the peak area is expected to be proportional to the extent of solubilisation:

11 $$A({c}_{{\rm{S}}}^{{\rm{S}}{\rm{A}}{\rm{T}}}\le {c}_{{\rm{S}}}\le {c}_{{\rm{S}}}^{{\rm{S}}{\rm{O}}{\rm{L}}})=f{c}_{{\rm{L}}}\frac{{c}_{{\rm{S}}}-{c}_{{\rm{S}}}^{{\rm{S}}{\rm{A}}{\rm{T}}}}{{c}_{{\rm{S}}}^{{\rm{S}}{\rm{O}}{\rm{L}}}-\,{c}_{{\rm{S}}}^{{\rm{S}}{\rm{A}}{\rm{T}}}}$$

Here, the last term on the right-hand side reflects the fraction of solubilised lipid as given by the lever rule^20, 21^.

Pairs of ${c}_{{\rm{S}}}^{{\rm{SAT}}}$ and ${c}_{{\rm{S}}}^{{\rm{SOL}}}$ values at a given lipid concentration were obtained by analysing the areas derived from the corresponding ^31^P NMR signals in terms of equations 9–11^16–18^. In addition to such local fits considering only one lipid concentration at a time, peak areas measured at four different lipid concentrations were globally fitted with equations 9–11 in order to obtain the best-fit ${R}_{{\rm{S}}}^{{\rm{b}},{\rm{SAT}}}$ and ${R}_{{\rm{S}}}^{{\rm{m}},{\rm{SOL}}}$ values. 95% confidence intervals were derived by nonlinear least-squares fitting in Excel spreadsheets, as detailed elsewhere^22^.

## Experimental Section

### Materials

DMPC and POPC were kind gifts from Lipoid (Ludwigshafen, Germany). SMA(2:1) (hydrolysed from styrene/maleic anhydride (2:1), tradename Xiran SZ30010) and SMA(3:1) (Xiran SL25010 S25) copolymer solutions were kind gifts from Polyscope (Geleen, Netherlands). DIBMA (Sokalan CP 9) was kindly provided by BASF (Ludwigshafen, Germany). D_2_O was purchased from Deutero (Kastellaun, Germany) and NaCl from VWR (Darmstadt, Germany). 85% (*w*/*v*) H_3_PO_4_ in D_2_O and Na_2_HPO_4_ were from Sigma–Aldrich (Steinheim, Germany), and Coomassie Brilliant Blue G250, NaH_2_PO_4_, ethylenediamine tetraacetic acid (EDTA), sodium dodecyl sulphate (SDS), tris(hydroxymethyl)aminomethane (Tris), and Tris–HCl were from Carl Roth (Karlsruhe, Germany). All chemicals were purchased in the highest purity available.

### Determination of copolymer refractive index increments

In order to measure copolymer concentrations, we modified a procedure based on a published protocol^14^. To this end, we precipitated 5 mL of a commercial SMA(2:1), SMA(3:1), or DIBMA solution by adding 3 mL of 4 M HCl and washed the pellets 4 times with 50 mL triple-distilled water. After each washing step, the polymer was pelleted by centrifugation at 8000 *g* for 15 min, and the supernatant was discarded. Washed pellets were resuspended in 3 mL of 0.5 M NaOH followed by a second precipitation and washing procedure as described above. Pellets were directly (i.e., without resuspension in NaOH^14^) aliquoted and lyophilised for at least 24 h using an Alpha 2–4 LSCplus (Martin Christ, Osterode am Harz, Germany). After lyophilisation, dried polymer powders were resuspended in 100 mM NaOH to yield polymer concentrations of 1% (*w*/*v*). Refractive index (RI) values were measured on an Abbemat 500 refractometer (Anton Paar, Graz, Austria) for dilution series comprising 5–10 polymer concentrations to determine RI increments (cf. Table 1).

**Table 1:** Molar RI increments (d*n*/d*c*), specific RI increments (d*n*/d*ρ*), number-average molar masses (*M*_*n*_), mass-average molar masses (*M*_*w*_), dispersities (*M*_*w*_/*M*_*n*_), molar extinction coefficients at 260 nm, *ε*_260_, and specific extinction coefficients at 260 nm, *µ*_260_, of nanodisc-forming polymers.

	**SMA(2:1)**	SMA(3:1)*	DIBMA**
**d** ***n*** **/d** ***c*** (L mol^−1^)	0.53	0.80	1.35
**d** ***n*** **/d** ***ρ*** (L kg^−1^)	0.20	0.20	0.16
***M***_***n***_ (kg mol^−1^)	2.7	4.0	8.4
***M***_***w***_ (kg mol^−1^)	7.0	10.0	15.3
***M***_***w***_ **/** ***M***_***n***_	2.60	2.50	1.82
***ε***_**260**_ (L (mol cm)^−1^)	4121	6989**	234
***µ***_**260**_ (L (kg cm)^−1^)	1526	1747	28

^*^Recalculated from Cuevas Arenas *et al*.^17^ using the modified protocol described here. **Taken from Oluwole *et al*.^18^.

### Preparation of SMA(2:1) stock solutions

SMA(2:1) has a styrene/maleic acid molar ratio of 2.2:1, a mass-average molar mass of *M*_*w*_ = 7.0 kg mol^−1^, a number-average molar mass of *M*_*n*_ = 2.7 kg mol^−1^, and, thus, a dispersity of *M*_*w*_/*M*_*n*_ = 2.6. Stock solutions of SMA(2:1) for vesicle solubilisation assays were prepared as described previously for SMA(3:1)^17^ and DIBMA^18^. Briefly, 3-mL aliquots of commercial SMA(2:1) solution were dialysed against 0.5 L Tris (50 mM Tris, 200 mM NaCl, pH 7.4 or 8.3) or phosphate buffer (50 mM Na_2_HPO_4_/NaH_2_PO_4_, 200 mM NaCl, pH 6.4) in 5-mL QuixSep dialysers (Membrane Filtration Products, Seguin, USA) using Spectra/Por 3 dialysis membranes with a molar-mass cutoff of 3.5 kg mol^−1^ (Spectrum Laboratories, Rancho Dominguez, California, USA). Dialysis was performed for 36 h at room temperature under gentle stirring with buffer exchange after 16 h. Dialysed stock solutions were sterile-filtered using 0.22-µm poly(vinylidene fluoride) syringe filters (Carl Roth, Karlsruhe, Germany), and final SMA(2:1) concentrations were determined refractometrically using the d*n*/d*c* value determined as described above (cf. Table 1). The concentrations of all polymers are reported on the basis of their respective number-average molar masses (cf. Table 1).

### Vesicle preparation

Lipid powders were suspended in either Tris buffer (50 mM Tris, 200 mM NaCl, pH 7.4 or 8.3) or phosphate buffer (50 mM Na_2_HPO_4_/NaH_2_PO_4_, 200 mM NaCl, pH 6.4) to final lipid concentrations of 30–45 mM. Lipid suspensions were vortexed for 10 min prior to 35-fold extrusion through two stacked polycarbonate membranes with a nominal pore diameter of 100 nm. DMPC was extruded at 30 °C using a block-heated Mini-Extruder (Avanti, Alabama, USA) and POPC at 20 °C using a LiposoFast extruder (Avestin, Ottawa, Canada). Unimodal particle size distributions were confirmed by DLS (see below), yielding hydrodynamic vesicle diameters of ~150 nm.

### ^31^P NMR spectroscopy

Samples containing 2.5, 5.0, 7.5, or 10.0 mM lipid and 0–4 mM SMA(2:1) (corresponding to 0–1.1% (*w*/*v*) copolymer) were prepared from stock solutions in Tris buffer (pH 7.4). 10% D_2_O (*v*/*v*) was included in the sample buffer to provide a lock signal. Samples were incubated for at least 16 h at 30 °C for DMPC or room temperature for POPC. NMR measurements were carried out at 30 °C for DMPC or 25 °C for POPC on an Avance 400 spectrometer (Bruker Biospin, Rheinstetten, Germany) operating at a ^31^P resonance frequency of 162 MHz using a 5-mm broadband inverse probe. 256 scans were acquired with an inverse-gated decoupling sequence using an acquisition time of 1.6 s, a sweep width of 9746 Hz, and a relaxation delay of 6 s. Data were multiplied by an exponential function with a line-broadening factor of 1.0 Hz before Fourier transformation. Chemical shifts were referenced to 85% (*w*/*v*) H_3_PO_4_ in D_2_O as external standard at 0 ppm. Peaks were integrated using the software Bruker Topspin 3.2.

### Dynamic light scattering

Samples containing 6 mM lipid in the form of LUVs and 0–3.1 mM SMA(2:1) (corresponding to 0–0.8% (*w*/*v*) copolymer) in either Tris buffer (pH 7.4 or 8.3) or phosphate buffer (pH 6.4) were incubated for at least 16 h at 30 °C for DMPC or room temperature for POPC. DLS measurements were performed on a Zetasizer Nano S90 (Malvern Instruments, Malvern, UK) working with a 633-nm He–Ne laser and a detection angle of 90°. Samples were thermostatted for 2 min at 30 °C for DMPC or 25 °C for POPC before measurements were performed in a 45-µL quartz glass cuvette with a cross-section of 3 mm × 3 mm (Hellma Analytics, Müllheim, Germany). Each sample was measured twice: firstly, with the attenuator position automatically optimised for determination of size distributions and, secondly, with the attenuator set to the maximum in order to ensure comparability of total scattering intensities. The effects of different NaCl concentrations and buffer components on the viscosity and RI of the solvent were accounted for during data analysis. Autocorrelation functions were fitted using a non-negatively constrained least-squares function^23^ to yield intensity-weighted particle size distributions and by cumulant analysis^24^ to obtain *z*-average particle diameters and associated polydispersity indices (PDIs). Distribution widths of *z*-average diameters, *σ*, were calculated as *σ* = $\,\sqrt{{\rm{PDI}}}\,z$.

### Differential scanning calorimetry

Samples containing 5 mM DMPC and 0–5 mM SMA(2:1) (corresponding to 0–1.4% (*w*/*v*) copolymer) in Tris buffer (pH 7.4) were incubated at 30 °C for 16 h prior to experiments. The sample and reference cells were filled with buffer and were repeatedly heated and cooled at a rate of 30 °C h^−1^ before the buffer in the sample cell was replaced with sample. Apart from the first upscan, successive heating and cooling scans, which were also performed at a rate of 30 °C h^−1^, overlaid very closely. Data were averaged, blank-subtracted, and normalised against the molar amount of DMPC in the sample using the software MicroCal Origin 7.0 (OriginLab, Northampton, USA). The melting temperature, *T*_m_, was taken as the temperature at which the excess molar isobaric heat capacity, Δ*C*_*p*_, reached a maximum.

### Solubilisation of native *E. coli* membranes

*E. coli* BL21(DE3) cells were transformed with an empty pET-24 vector and selected by kanamycin resistance. After incubation in lysogeny broth overnight at 37 °C under permanent agitation, cells were harvested by centrifugation and washed twice with saline (154 mM NaCl). Cell pellets were resuspended in a 10-fold volume of ice-cold buffer (50 mM Tris, 2 mM EDTA, 200 mM NaCl, pH 7.4) and ultrasonicated twice for 10 min in an S-250 A sonifier (Branson Ultrasonics, Danbury, USA). The lysate was further centrifuged for 30 min at 1000 *g* and 4 °C. The supernatant was ultracentrifuged for 1 h at 100,000 *g* and 4 °C and subjected to 7 buffer washing steps to remove soluble proteins. Membrane pellets were resuspended in buffer (50 mM Tris, 200 mM NaCl, pH 7.4) to a final concentration of 42.5 mg mL^−1^ and treated with 10 mM (0.5% (*w*/*v*)) DDM, 9.3 mM (2.5% (*w*/*v*)) SMA(2:1), 6.3 mM (2.5% (*w*/*v*)) SMA(3:1), 3.0 mM (2.5% (*w*/*v*)) DIBMA, or buffer. Polymer-containing samples were incubated for 16 h at 20 °C with gentle agitation and subsequently subjected to ultracentrifugation for 1 h at 100,000 *g* and 4 °C. The supernatant containing solubilised membrane proteins was analysed by sodium dodecyl sulphate polyacrylamide gel electrophoresis (SDS-PAGE). To avoid band smearing caused by the presence of polymers^14^, solubilised fractions were precipitated with CH_3_OH/CHCl_3_/H_2_O in a mixing ratio of 4:1:3 (*v*/*v*/*v*)^25^. Briefly, to a 100-μL aliquot of ice-cold sample, we successively added 400 μL ice-cold CH_3_OH, 100 μL ice-cold CHCl_3_, and 300 μL ice-cold water with thorough vortexing after each addition. The mixture was centrifuged for 2 min at 14,000 *g* and 4 °C. The upper, aqueous layer was removed, and 400 μL CH_3_OH was added before the sample was vortexed again. Precipitated proteins were pelleted by centrifugation for 1 min at 5000 *g* and another 5 min at 20,000 *g*, both at 4 °C. This two-step centrifugation was performed to make sure that the pellet completely sticks to the bottom rather than the sides of the centrifugation tube. CH_3_OH was carefully removed using a pipette. Residual organic solvent was allowed to evaporate under a chemical hood and was subsequently removed under high vacuum in a desiccator overnight. The dried pellet was resuspended in SDS buffer (106 mM Tris-HCl, 141 mM Tris, 2% (*w*/*v*) SDS, 10% (*w*/*v*) glycerol, 0.51 mM EDTA, 0.22 mM Coomassie Brilliant Blue G250, 0.175 mM Phenol Red, pH 8.5), boiled for 10 min under agitation, and subjected to SDS-PAGE.

## Results and Discussion

### Determination of copolymer concentrations by refractometry

We have recently shown that refractometry is a useful tool for determining the concentrations of SMA(3:1)^16, 17^ and DIBMA^18^ in aqueous solutions. Refractometry is particularly valuable for DIBMA, which contains no aromatic residues and, thus, no chromophores that would allow a straightforward quantification by UV absorbance^18^. Our previous protocols for determining the RI increments of SMA(3:1)^16, 17^ and DIBMA^18^ differed from one another in that the contribution of NaOH to the RI of the polymer stock solution was accounted for only in the case of DIBMA. Thus, to allow for quantitative comparisons among different polymers, we established a refined and general protocol for the refractometric quantification of polymer concentrations. As described in a recent protocol for preparing SMA(2:1) solutions^14^, we first washed and lyophilised the polymer solutions obtained from the manufacturer (cf. Experimental Section for details). The major departure from the standard procedure^14^ was that, rather than resuspending the polymer pellets in NaOH for lyophilisation after the last washing step, we directly lyophilised the pelleted polymer. This approach aimed at minimising the Na^+^ and Cl^−^ contents in the lyophilised polymer pellets, which otherwise would be difficult to control or quantify but would also contribute to the measured RI values. We then resuspended the lyophilised polymer in 100 mM NaOH and measured RI values at different concentrations of each polymer. The constant contribution of NaOH to the RI signal was accounted for by subtracting the RI value of a 100 mM NaOH blank. For each polymer, both molar and specific RI increments, d*n*/d*c* and d*n*/d*ρ*, were obtained from the slopes of the plots of RI against molar and mass concentrations, *c* and *ρ*, respectively. Table 1 summarises the d*n*/d*c* and d*n*/d*ρ* values thus determined along with the corresponding number-average molar masses, *M*_*n*_, mass-average molar masses, *M*_*w*_, and dispersities, *M*_*w*_/*M*_*n*_, of the three polymers. To provide a comprehensive overview of the physicochemical properties of the three polymers that can be used to determine their concentrations, Table 1 lists also their molar extinction coefficients at 260 nm, *ε*_260_, and specific extinction coefficients at 260 nm, *µ*_260_. Note that the extinction coefficients of DIBMA are shown for completeness only, as they are of limited use for concentration determination (see above).

Unsurprisingly, d*n*/d*c* increases with increasing molar mass of the polymer, whereas the d*n*/d*ρ* values are identical for SMA(2:1) and SMA(3:1) but significantly lower for DIBMA. The d*n*/d*c* value of SMA(3:1) reported in Table 1 is 28% lower than the value previously estimated without the above-mentioned correction for the presence of inorganic ions^16, 17^. Thus, use of the d*n*/d*c* value determined here results in somewhat higher SMA(3:1) concentrations than those reported previously, which does not, however, affect any of the major conclusions drawn from these earlier studies^16, 17^.

### Solubilisation of saturated phospholipids by SMA(2:1)

**Figure 1:**
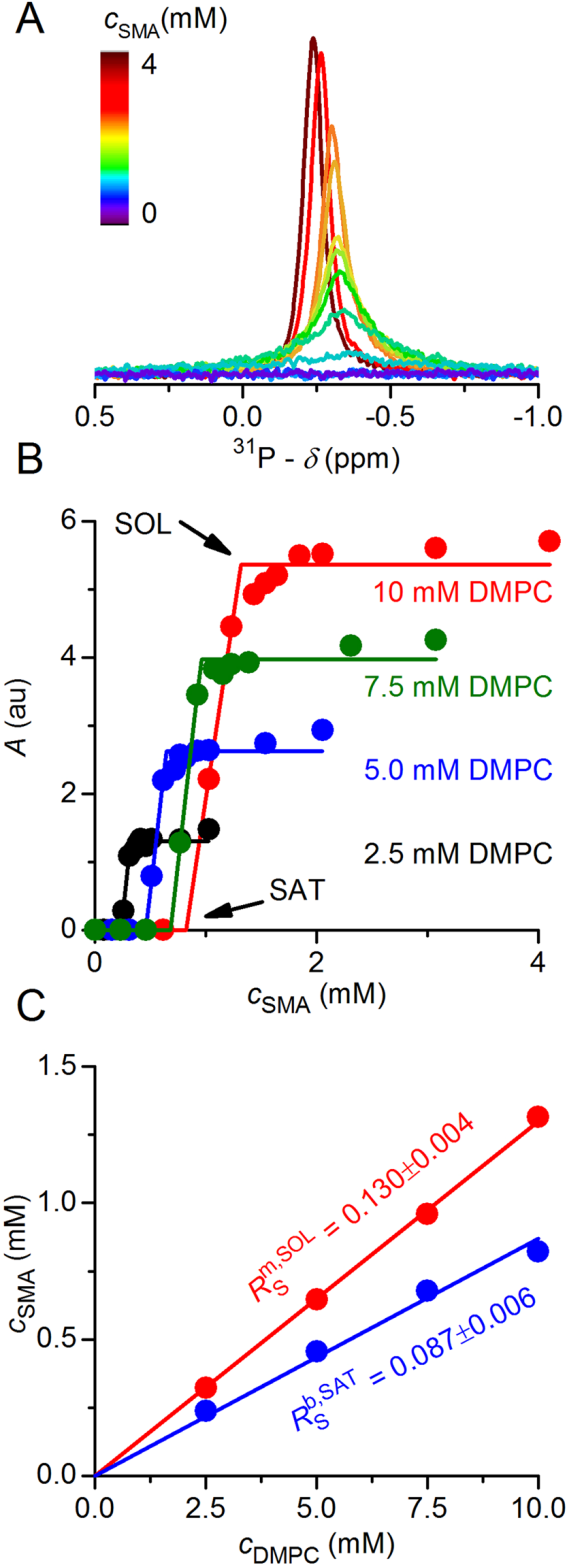
Solubilisation of DMPC vesicles by SMA(2:1) at 30 °C as monitored by ^31^P NMR. (**A**) NMR spectra of 10 mM DMPC initially present in the form of LUVs upon exposure to increasing concentrations of SMA(2:1). (**B**) Peak areas, *A*, at four different DMPC concentrations as functions of SMA(2:1) concentration, showing experimental data (circles) and global fits (lines) according to equations 9–11. The slight increase in *A* upon complete solubilisation is due to a decrease in nanodisc size with increasing SMA(2:1) concentration, thus resulting in sharper peaks that are better resolved from the baseline, as seen in A. (**C**) Phase diagram of DMPC/SMA(2:1) at 30 °C showing the onset (saturation; SAT) and completion (solubilisation; SOL) of solubilisation. Shown are pairs of ${c}_{{\rm{S}}}^{{\rm{SAT}}}\,$and ${c}_{{\rm{S}}}^{{\rm{SOL}}}$ (circles) obtained from breakpoints derived from local fits in (**B**) and global fits (solid lines).

**Figure 2:**
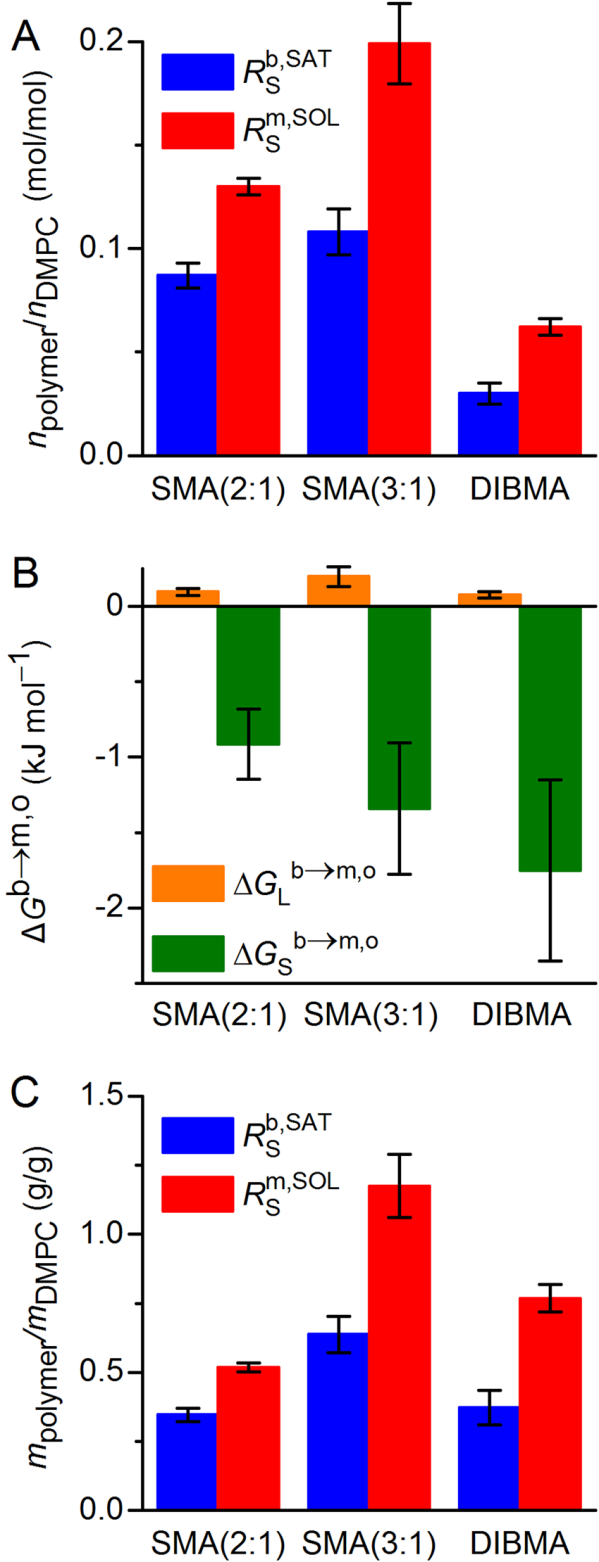
Thermodynamic parameters characterising the solubilisation of DMPC vesicles at 30 °C by the three copolymers compared in this study. (**A**) SAT and SOL phase boundaries of SMA(2:1), SMA(3:1), and DIBMA based on polymer/lipid *molar* ratios. (**B**) Transfer free energies of lipid and copolymers, ${\rm{\Delta }}{G}_{{\rm{L}}}^{{\rm{b}}\to {\rm{m}},{\rm{o}}}$ and ${\rm{\Delta }}{G}_{{\rm{S}}}^{{\rm{b}}\to {\rm{m}},{\rm{o}}}$, respectively, as derived from phase boundaries in (**A**). (**C**) SAT and SOL phase boundaries of SMA(2:1), SMA(3:1), and DIBMA based on copolymer/lipid *mass* ratios. Error bars denote 95% confidence intervals, roughly corresponding to ±2 standard deviations.

To quantify the solubilisation of lipid-bilayer vesicles by SMA(2:1) under equilibrium rather than kinetically controlled conditions, we used ^31^P NMR spectroscopy to follow the solubilisation of DMPC LUVs at 30 °C. This temperature is well above the main phase-transition temperature, *T*_m_, of DMPC, so that the lipid bilayer was always in the liquid–crystalline (i.e., fluid) state. In the absence of copolymer, the NMR signal of large, slow-tumbling vesicles was broadened beyond detection (Fig. 1A). Addition of SMA(2:1) at concentrations above the saturation (SAT) boundary resulted in the emergence of an isotropic peak, thus indicating the formation of smaller, fast-tumbling lipid particles^16–18^. Beyond this point, the peak area increased linearly with the concentration of SMA(2:1), until a plateau reflecting the completion of solubilisation was reached at the solubilisation (SOL) boundary. At each lipid concentration tested, the NMR peak area reflected such a three-stage solubilisation behaviour with two breakpoints, namely, the SAT and SOL phase boundaries (Fig. 1B). The concentrations of SMA(2:1) at the SAT and SOL boundaries at four different DMPC concentrations yielded a phase diagram characterised by critical SMA(2:1)/DMPC molar ratios of ${R}_{{\rm{S}}}^{{\rm{b}},{\rm{SAT}}}$ = 0.087 ± 0.006 and ${R}_{{\rm{S}}}^{{\rm{m}},{\rm{SOL}}}$ = 0.130 ± 0.004, respectively (Fig. 1C). These ratios furnished Gibbs free-energy changes accompanying the vesicle-to-nanodisc transfer of ${\rm{\Delta }}{G}_{{\rm{L}}}^{{\rm{b}}\to {\rm{m}},{\rm{o}}}$ = (0.098 ± 0.023) kJ mol^−1^ and ${\rm{\Delta }}{G}_{{\rm{S}}}^{{\rm{b}}\to {\rm{m}},{\rm{o}}}$ = – (0.91 ± 0.23) kJ mol^−1^ for the lipid and the polymer, respectively (Fig. 2A).

### Comparison of equilibrium solubilisation efficiencies among copolymers

On the basis of the new data (Fig. 1) and earlier results^16–18^, we compared the equilibrium solubilisation efficiencies of SMA(2:1), SMA(3:1), and DIBMA toward DMPC LUVs in terms of their SAT and SOL phase boundaries and the Gibbs free-energy changes accompanying the vesicle-to-nanodisc transfer of the lipid and the polymers. The critical polymer/lipid *molar* ratios required for the onset and completion of solubilisation in the case of SMA(2:1) were significantly lower than for SMA(3:1) but higher than for DIBMA (Fig. 2A). Since the average molar masses of the copolymers exceed that of the lipid by a factor of 4–12, comparisons based on polymer/lipid *molar* ratios should be taken with caution. Notwithstanding this caveat, knowledge of the polymer/lipid ratio required for solubilising a given amount of lipids is instructive not only for practical purposes but also for thermodynamic considerations relying on the Gibbs free energies deduced from ${R}_{{\rm{S}}}^{{\rm{b}},{\rm{SAT}}}\,\,$and ${R}_{{\rm{S}}}^{{\rm{m}},{\rm{S}}{\rm{O}}{\rm{L}}}$ (Fig. 2B). We found ${\rm{\Delta }}{G}_{{\rm{S}}}^{{\rm{b}}\to {\rm{m}},{\rm{o}}}$, which drives nanodisc formation, to be most favourable for DIBMA and least favourable for SMA(2:1). In the case of the two SMA copolymers, insertion of their planar phenyl moieties into the lipid bilayer is expected to make a substantial contribution to the adsorption of the polymers onto the membrane^26^. This could render the membrane-adsorbed state of SMA(2:1) relatively stable, thereby reducing the absolute value of ${\rm{\Delta }}{G}_{{\rm{S}}}^{{\rm{b}}\to {\rm{m}},{\rm{o}}}$. By contrast, the bulky, branched neopentyl moieties of DIBMA are less easy to intercalate among the lipid acyl chains, which may favour the nanodisc-surrounding over the membrane-adsorbed state.

**Figure 3:**
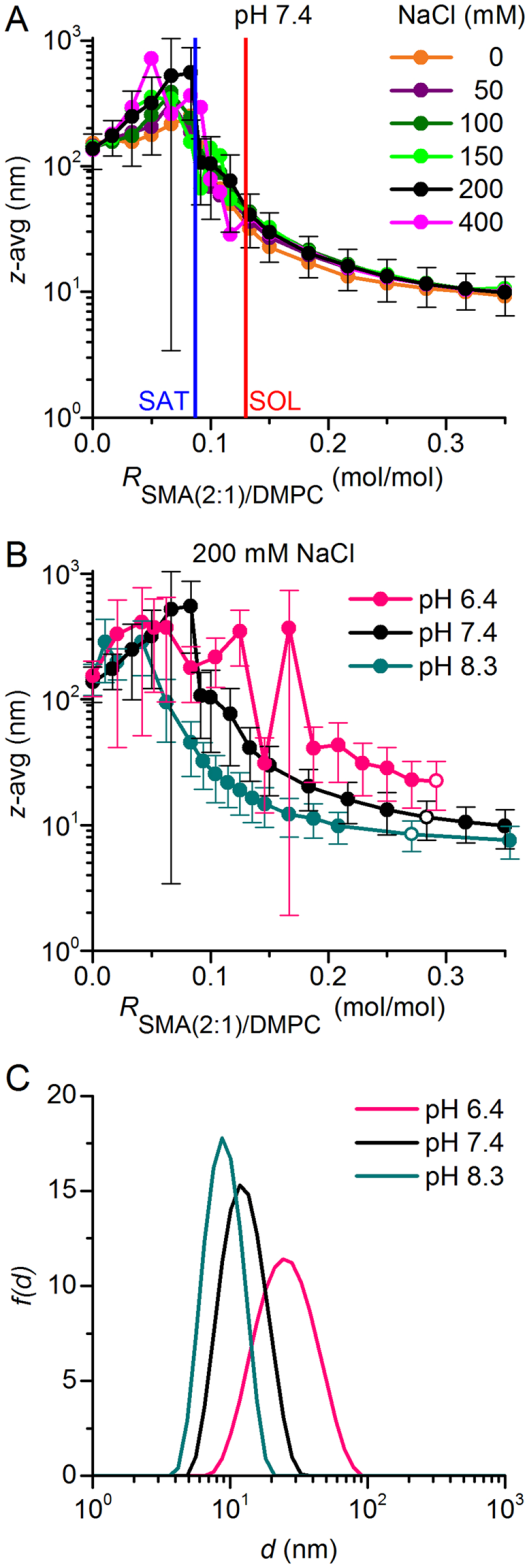
Solubilisation of DMPC vesicles by SMA(2:1) at 30 °C in the presence of different NaCl concentrations and pH values as monitored by DLS. (**A**) *z*-Average diameters as functions of SMA(2:1)/DMPC molar ratio at pH 7.4 and increasing NaCl concentrations. Vertical lines indicate the SAT and SOL boundaries derived from NMR in the presence of 200 mM NaCl (Fig. 1C). (**B**) *z*-Average diameters as functions of SMA(2:1)/DMPC molar ratio at 200 mM NaCl and different pH values. Error bars denote peak distribution widths as given by *σ* =$\,\sqrt{{\rm{PDI}}}\,z.$ (**C**) Intensity-weighted particle size distributions, *f*(*d*), as functions of pH at similar, completely solubilising SMA(2:1)/DMPC molar ratios as indicated by open circles in (**B**).

As noted above, comparisons among the three polymers on a *molar* basis need to be interpreted with caution, since SMA(2:1) (*M*_*n*_ = 2.7 kg mol^−1^) is smaller than SMA(3:1) (*M*_*n*_ = 4.0 kg mol^−1^) and much smaller than DIBMA (*M*_*n*_ = 8.4 kg mol^−1^). To account for these differences in molecular size, we converted the phase boundaries from *molar* ratios (i.e., with units of mol/mol) to *mass* ratios (i.e., g/g). On this *mass* ratio scale, SMA(2:1) is the most efficient solubiliser, followed by DIBMA and SMA(3:1) (Fig. 2C). The observation that SMA(2:1) has a higher equilibrium lipid-solubilisation efficiency than SMA(3:1) is interesting in the light of a recent study^13^ reporting that, among several SMA variants, SMA(2:1) is the preferred choice for solubilising membrane proteins. Specifically, three membrane proteins of different sizes, topologies, and functions have been shown to be most efficiently solubilised by SMA(2:1) in terms of the total amounts, purities, and functionalities of the extracted proteins^13^. Moreover, our equilibrium solubilisation efficiencies correlate with the finding that SMA(2:1) is the most efficient copolymer as regards the solubilisation kinetics of lipid vesicles^15^. It should be noted that such a correlation between equilibrium and kinetic results is not trivial, as will be discussed in more detail below (cf. Figures 3B and 5F).

### Effects of ionic strength and pH on the equilibrium solubilisation efficiency of SMA(2:1)

It has been shown that both ionic strength^26^ and pH^15^ modulate the kinetics of membrane solubilisation by SMA(2:1). However, no data are presently available that report on the effects of these two solution properties on the equilibrium of SMA(2:1)-mediated lipid-bilayer solubilisation. Therefore, we titrated DMPC with SMA(2:1) in the presence of 0–400 mM NaCl at pH 7.4 to study the influence of ionic strength and, in another set of experiments, kept the NaCl concentration at 200 mM and monitored the solubilisation equilibrium at pH values of 6.4, 7.4, and 8.3. Hydrodynamic particle sizes monitored by DLS (Fig. 3A) furnished two major observations when the ionic strength of the buffer was varied: (i) Under subsolubilising conditions, where polymer-coated vesicles tend to aggregate^16–18^, the apparent particle diameters increased with NaCl concentration. This could be explained by stronger salt screening of the repulsive Coulomb forces that must act among vesicles carrying polyanionic copolymer chains, thus facilitating vesicle aggregation. However, it is important to point out that the increase in particle size with ionic strength suggested by DLS might, at least in part, be only apparent. The calculation of particle sizes from diffusion coefficients is based on the assumption that the particles do not interact with each other. At low ionic strength, this is a poor assumption; in fact, interparticle repulsion then will lead to an overestimation of the diffusion coefficient and an underestimation of the particle size. Hence, the hydrodynamic diameters obtained at elevated ionic strength might reflect the true particle sizes more closely than those determined in the presence of low salt concentrations. (ii) Under conditions of complete solubilisation, there were no differences in nanodisc size among different NaCl concentrations, with a smooth decrease in hydrodynamic diameter^17^ down to ~10 nm at SMA(2:1)/DMPC molar ratios in excess of ~0.3. Hence, the solubilisation of DMPC LUVs was not significantly different at low ionic strength as compared with higher salt concentrations. In particular, no shift in the SOL boundary was detected, as one might have expected from the fact that decreasing ionic strength slows down the solubilisation process^26^.

Contrary to the lack of influence of ionic strength on DMPC solubilisation efficiency and nanodisc size, we observed a pronounced effect upon varying the pH value at a constant salt concentration of 200 mM NaCl (Fig. 3B,C). At pH 6.4, substantially higher concentrations of SMA(2:1) were required for solubilisation than at pH 7.4, where solubilisation was, in turn, less efficient than at pH 8.3. This equilibrium behaviour is in stark contrast with the kinetics of DMPC solubilisation by SMA(2:1), which becomes slower with increasingly alkaline pH^15^. The latter observation has been attributed to the effect of pH on the conformation of the copolymer^15^; accordingly, SMA(2:1) becomes more charged and less hydrophobic with increasing pH, which results in a more extended chain conformation stabilised by electrostatic repulsion, thereby reducing the driving force for membrane adsorption and solubilisation. The present thermodynamic findings, however, suggest a different, more nuanced picture of the important role of pH-dependent conformational properties of SMA(2:1) in the lipid-solubilisation process: At elevated pH, the reduced effective hydrophobicity of SMA(2:1) appears to slow down the solubilisation of phospholipids from vesicles; once solubilisation has occurred, however, the more extended conformation of the copolymer chains allows for a more efficient encapsulation of solubilised lipids, thereby lowering the minimum amount of SMA(2:1) required for complete solubilisation. In summary, the solubilisation of DMPC LUVs by SMA(2:1) is slower but thermodynamically more efficient at moderately alkaline than at neutral or slightly acidic pH values.

### Influence of SMA(2:1) on the gel-to-fluid phase transition of phospholipids

Owing to its main phase-transition temperature of *T*_m_ ≈ 24 °C, DMPC lends itself for analysing thermotropic lipid phase transitions and, thus, membrane-perturbing effects of amphiphilic copolymers with the aid of DSC. Previous DSC studies of DMPC in nanodiscs bounded by SMA(2:1)^5^, SMA(3:1)^4, 18^, or DIBMA^18^ have used various buffer conditions and, more critically, different copolymer/lipid ratios, which impedes straightforward comparisons among the three copolymers. Therefore, we investigated the concentration-dependent effects of SMA(2:1) on the thermotropic phase behaviour of DMPC in more detail and compared them with those of SMA(3:1) and DIBMA under identical conditions.

**Figure 4:**
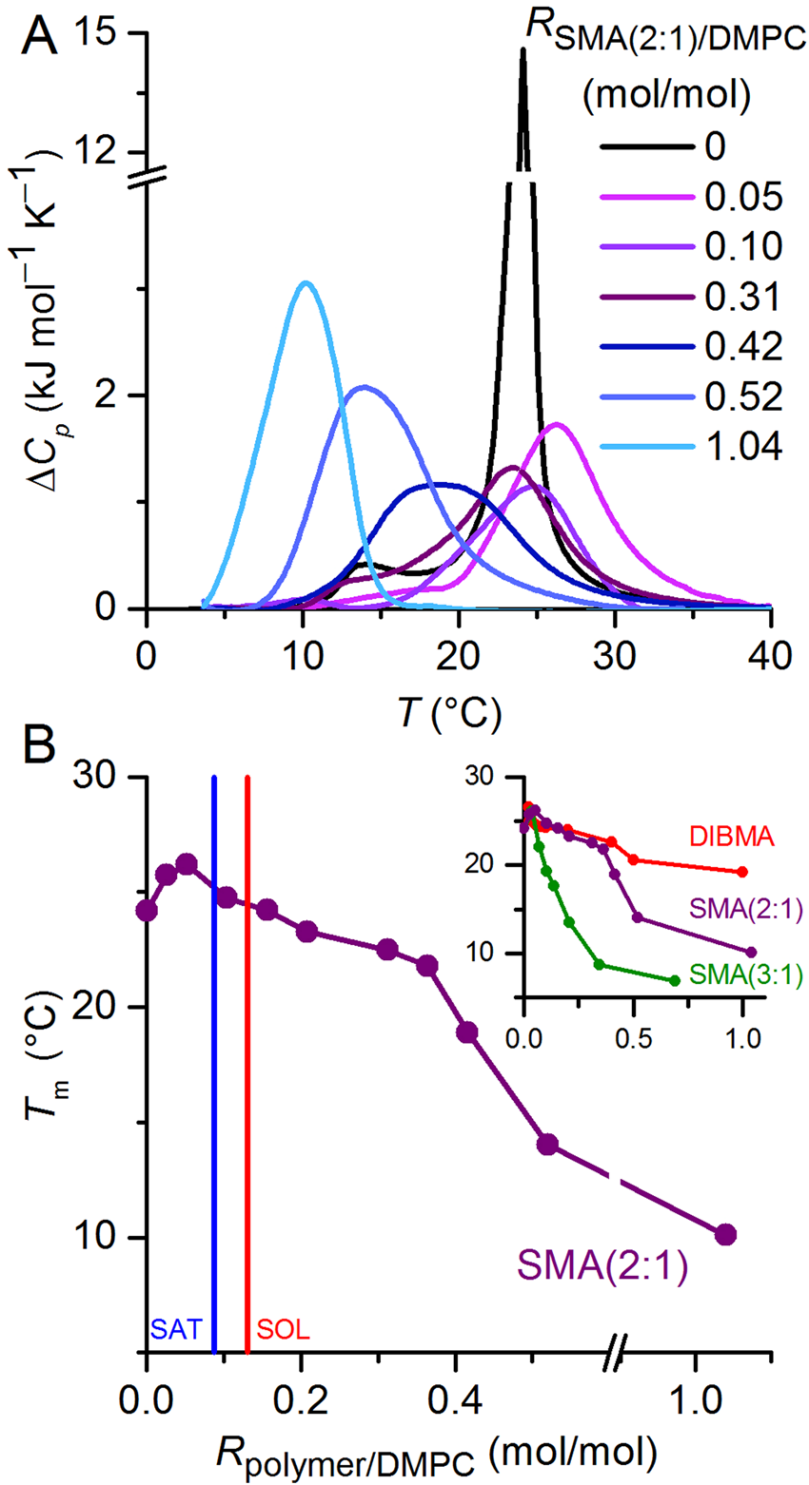
Thermotropic phase behaviour of DMPC upon solubilisation by SMA(2:1) at pH 7.4. (**A**) DSC thermograms showing excess molar isobaric heat capacities, ∆*C*_*p*_, of 5 mM DMPC initially present in the form of LUVs upon exposure to increasing concentrations of SMA(2:1). (**B**) Gel-to-fluid phase transition temperature, *T*_m_, of 5 mM DMPC in the presence of increasing concentrations of SMA(2:1), SMA(3:1), or DIBMA. SMA(3:1)/DMPC molar ratios were recalculated from Oluwole et al.^18^ using the d*n*/d*c* value determined in this work (cf. Table 1). Vertical lines in the main panel indicate the SAT and SOL boundaries of SMA(2:1) derived from NMR at 30 °C (Fig. 1C). The same *T*_m_ values are shown as functions of polymer/DMPC *mass* ratios in Supplementary Figure 1.

In the absence of copolymer, a highly cooperative gel-to-fluid phase transition typical of DMPC LUVs was observed at 24 °C (Fig. 4A). In the presence of SMA(2:1), the thermograms were broadened, and the peak height decreased by a factor of ~10. After a slight increase in *T*_m_ at polymer/lipid ratios below ${R}_{{\rm{S}}}^{{\rm{b}},{\rm{SAT}}}$, there was only a marginal downshift in *T*_m_ to ~23 °C upon complete solubilisation of DMPC vesicles by SMA(2:1) (Fig. 4B). Above ${R}_{{\rm{S}}}^{{\rm{m}},{\rm{SOL}}}$, *T*_m_ monotonically decreased with increasing SMA(2:1) concentration, with a pronounced kink at an SMA(2:1)/DMPC molar ratio of ∼0.4, that is, well within the fully solubilised range. The initial increase in *T*_m_ reflects a stabilisation of the gel over the fluid phase, possibly caused by partial dehydration of the membrane surface upon adsorption of the copolymer. Once solubilisation is complete, *T*_m_ decreases because the lipid molecules tend to pack more loosely in nanodiscs than in vesicles, which might be due to the intercalation of the copolymer’s phenyl moieties^4, 5^. Among the three polymers, SMA(3:1) had the most drastic effect on *T*_m_ (Fig. 4B, inset), which suggests a stronger perturbation of lipid acyl-chain packing by this copolymer as compared with SMA(2:1), which has a lower styrene content, and DIBMA, which contains no aromatic groups at all.

The DSC data also highlight two significant differences between polymer-based nanodiscs on the one hand and nanodiscs bounded by membrane scaffold proteins (MSPs) on the other hand: First, lipid-bilayer nanodiscs surrounded by amphiphilic copolymers generally exhibit reduced *T*_m_ values indicative of a less densely packed acyl-chain core, whereas MSP nanodiscs have slightly increased *T*_m_ values as compared with vesicular membranes^27^. Second, unlike in the case of MSP-bounded nanodiscs^27^, repeated DSC scans of the same samples were found to be highly reproducible for all three types of polymer-bounded nanodiscs (data not shown), attesting to their pronounced thermal stability.

In addition to changes in *T*_m_, the transition peak of SMA(2:1)/DMPC nanodiscs monotonously broadened with increasing polymer concentration up to a copolymer/lipid molar ratio of ∼0.4 (Fig. 4A). Such broadening has been reported for MSP nanodiscs^27^ as well as for nanodiscs surrounded by SMA(2:1)^5^, SMA(3:1)^4, 18^, and DIBMA^18^ and is readily explained by a decrease in the number of lipid molecules in the “cooperative unit” in nanodiscs as compared with LUVs. In addition to this straightforward explanation, it should be kept in mind that the thermotropic phase behaviour of DMPC molecules that are in close vicinity to the polymer rim most likely differ drastically from that of “bulk” lipids. In an extreme scenario, such lipid molecules could be fluidised even at the lowest experimental temperatures and, then, would be excluded from the observed phase transition. If the thermotropic phase transition of peripheral lipids is not abolished but merely shifted to lower or higher temperatures than that of lipid molecules in the nanodisc centre, the transition peak will be further broadened.

### Solubilisation of unsaturated phospholipids by SMA(2:1)

To more closely mimic biological membranes containing unsaturated phospholipids, we also investigated the solubilisation of POPC LUVs by SMA(2:1). POPC is an unsaturated zwitterionic phospholipid naturally present in most eukaryotic and some prokaryotic cells^28^, which therefore is often used as a model membrane lipid for *in vitro* research.

**Figure 5:**
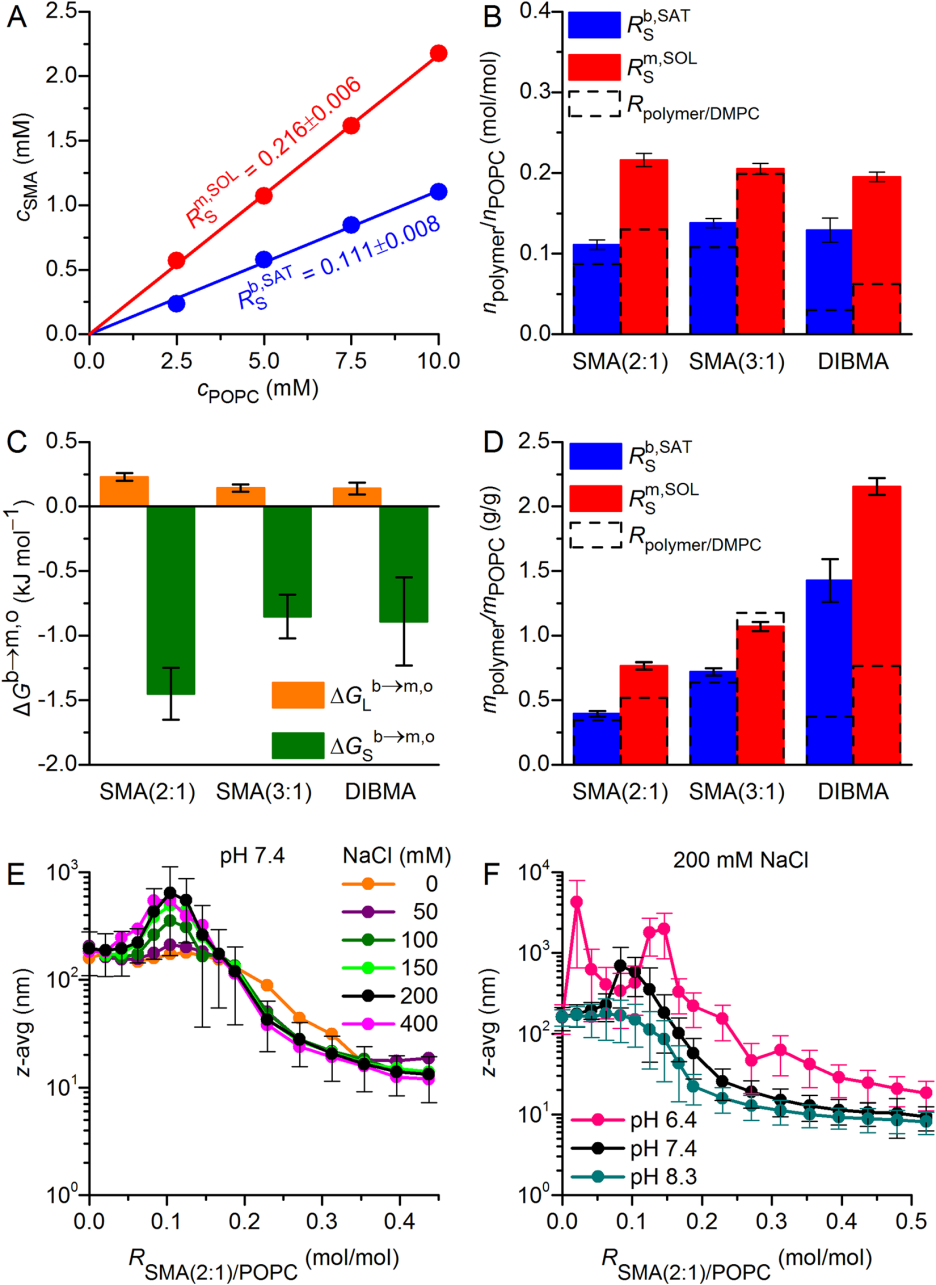
Solubilisation of POPC vesicles by SMA(2:1) at 25 °C as monitored by ^31^P NMR and DLS. (**A**) Phase diagram of POPC/SMA(2:1) showing the onset (saturation; SAT) and completion (solubilisation; SOL) of solubilisation. Shown are pairs of ${c}_{{\rm{S}}}^{{\rm{b}},{\rm{SAT}}}\,$and ${c}_{{\rm{S}}}^{{\rm{m}},{\rm{SOL}}}$ (circles) obtained from breakpoints derived from local fits and global fits (solid lines). (**B**) SAT and SOL phase boundaries of SMA(2:1), SMA(3:1), and DIBMA based on copolymer/lipid *molar* ratios. For comparison, corresponding SAT and SOL phase boundaries determined for DMPC (Fig. 2A) are indicated as dashed bars. (**C**) Transfer free energies of lipid and copolymers, ${\rm{\Delta }}{G}_{{\rm{L}}}^{{\rm{b}}\to {\rm{m}},{\rm{o}}}$ and ${\rm{\Delta }}{G}_{{\rm{S}}}^{{\rm{b}}\to {\rm{m}},{\rm{o}}}$, respectively, as derived from phase boundaries in (**B**). (**D**) SAT and SOL phase boundaries of SMA(2:1), SMA(3:1), and DIBMA based on copolymer/lipid *mass* ratios. For comparison, corresponding SAT and SOL phase boundaries determined for DMPC (Fig. 2C) are indicated as dashed bars. (**E**) *z*-Average diameters as functions of SMA(2:1)/POPC molar ratio at different NaCl concentrations. (**F**) *z*-Average diameters as functions of SMA(2:1)/POPC molar ratio at pH values of 6.4, 7.4, and 8.3. Error bars denote peak distribution widths as given by *σ* =$\,\sqrt{{\rm{PDI}}}\,z$.

The pseudophase diagram of SMA(2:1) and POPC (Fig. 5A) reveals saturating and solubilising SMA(2:1)/POPC molar ratios of ${R}_{{\rm{S}}}^{{\rm{b}},{\rm{SAT}}}=0.111\pm 0.008$ and ${R}_{{\rm{S}}}^{{\rm{m}},{\rm{SOL}}}=0.216\pm 0.006$, respectively. Thus, both the SAT and the SOL boundaries of SMA(2:1) are higher for POPC than for DMPC. Comparing these values with those of SMA(3:1) and DIBMA on a *molar* concentration scale unveils only minor differences among their solubilisation efficiencies (Fig. 5B). This is because of compensating differences in the transfer Gibbs free energies of POPC and the three copolymers, as ${\rm{\Delta }}{G}_{{\rm{L}}}^{{\rm{b}}\to {\rm{m}},{\rm{o}}}$ is more unfavorable for SMA(2:1) than for SMA(3:1) and DIBMA, whereas ${\rm{\Delta }}{G}_{{\rm{S}}}^{{\rm{b}}\to {\rm{m}},{\rm{o}}}$ is more favorable for SMA(2:1) than for SMA(3:1) and DIBMA (Fig. 5C). Again, a different picture emerges when solubilisation efficiencies are compared on a *mass* concentration scale, which may be more relevant for many practical applications: on this scale, it is apparent that SMA(2:1) is the most powerful solubiliser of POPC bilayers, followed by SMA(3:1) and DIBMA (Fig. 5D). The most conspicuous result that emerges from a comparison of POPC and DMPC solubilisation thermodynamics (Fig. 5B,D; dashed) is that the two SMA copolymers are much less susceptible to the effect of chain unsaturation than is DIBMA. Presumably, the increased lateral pressure^29^ in the acyl-chain region of the lipid bilayer that is due to the presence of a double bond in POPC has a more detrimental effect on the insertion of the bulky aliphatic side chains of DIBMA than on the intercalation of the planar aromatic groups of SMA.

In contrast with the case of DMPC, the solubilisation efficiency of SMA(2:1) toward POPC was slightly impaired at very low ionic strength, that is, in the absence of additional NaCl (Fig. 5E). Under such conditions, the hydrodynamic particle diameter decreased more gradually than in the presence of higher NaCl concentrations. In general, accumulation of a highly negatively charged polymer on or within a membrane will be facilitated by higher ionic strengths because of electrostatic screening^26^. We hypothesise that, in the case of LUVs composed of the saturated phospholipid DMPC, adsorption and penetration of SMA(2:1) into the lipid bilayer are so strong that a decrease in ionic strength has no significant effect. By contrast, bilayer penetration should be more difficult in the case of POPC because of the increased lateral pressure in the acyl-chain region of the membrane^29^. Thus, polymer adsorption is weaker at low ionic strengths but becomes stronger as the electrostatic repulsion is screened at higher salt concentrations. For POPC, we found the same pH dependence as observed for DMPC, that is, the equilibrium solubilisation efficiency was lowest at pH 6.4 and highest at pH 8.3 (Fig. 5F).

### Solubilisation of native *E. coli* membranes by SMA(2:1)

**Figure 6:**
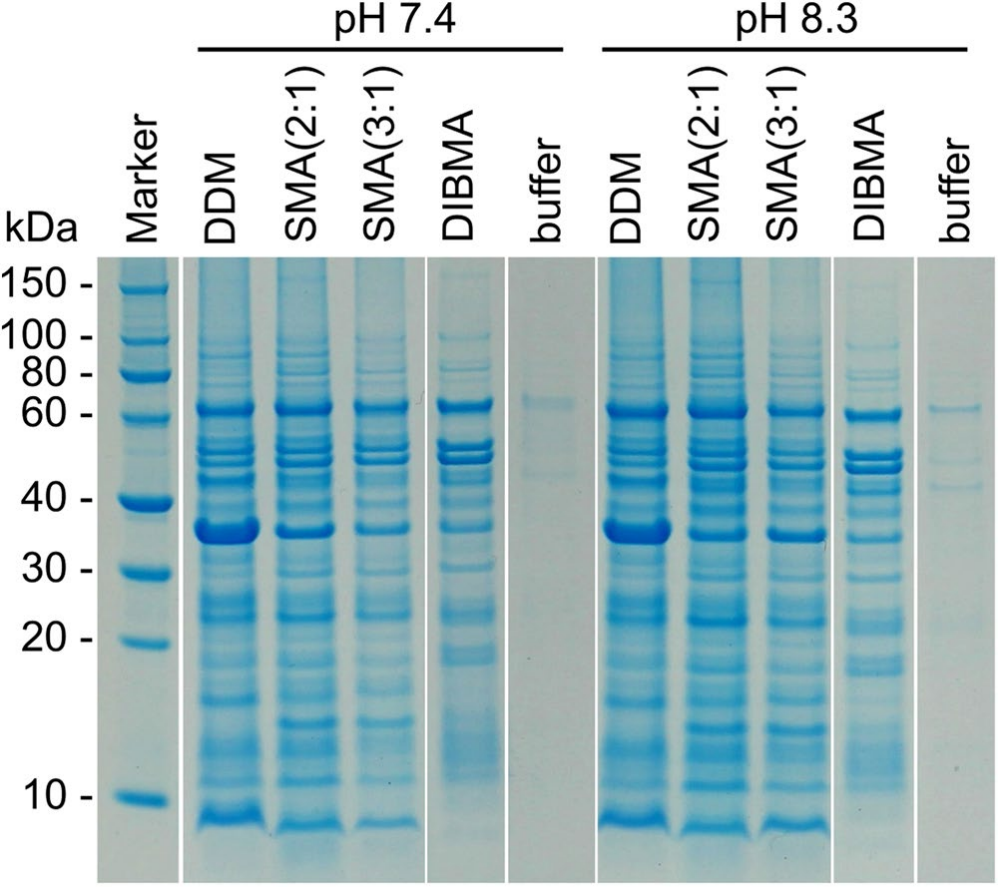
SDS-PAGE showing the solubilisation of *E. coli* BL21(DE3) membranes by 10 mM (0.5% (*w*/*v*)) DDM, 9.3 mM (2.5% (*w*/*v*)) SMA(2:1), 6.3 mM (2.5% (*w*/*v*)) SMA(3:1), or 3.0 mM (2.5% (*w*/*v*)) DIBMA. Buffer conditions were 50 mM Tris, 200 mM NaCl, 20 °C. Shown are the solubilised membrane-protein fractions after removal of cell debris, intrinsically soluble proteins, and unsolubilised material by serial centrifugation. Data for DDM, SMA(3:1), and DIBMA are reproduced from Oluwole et al.^18^ For clarity and conciseness, the gel was cropped as indicated. The full-length gel is presented in Supplementary Figure 2.

Both SMA(3:1) and DIBMA solubilise a broad range of membrane proteins directly from *E. coli* membranes but show different pH dependencies^18^. After evaluating the equilibrium efficiency of SMA(2:1) in solubilising model lipid vesicles, we were interested in testing its performance on native membranes, which represent chemically heterogeneous, protein-containing targets. While homogenous, well-defined model lipid vesicles enable a quantitative description of the thermodynamics of solubilisation, the extraction of membrane proteins from biological membranes depends not only on their lipid matrix, the composition of which is often poorly defined, but also on the types and contents of the many diverse protein constituents. In general, the solubilisation behaviour of model membranes by detergents is, therefore, not directly transferable to native membranes. This motivated us to extend our previous analysis of SMA(3:1) and DIBMA^18^ by determining the protein extraction yields of SMA(2:1) at pH 7.4 and 8.3 and compare them with those of SMA(3:1) and DIBMA under identical conditions (cf. Experimental Section for details). Briefly, we prepared *E. coli* membrane fragments, solubilised proteins, performed SDS-PAGE, and used densitometry to quantify the total amounts of protein extracted by each of the three polymers (Fig. 6).

At both pH values, the protein-solubilisation yield of SMA(2:1) amounted to >90% relative to that of the commonly used detergent *n*-dodecyl-β-d-maltopyranoside (DDM). This underlines the excellent performance of SMA(2:1) in solubilising native membranes, which affords protein yields 10–30% higher than those of SMA(3:1) and DIBMA. Additionally, we found that the solubilisation yield of SMA(2:1) was >10% higher at pH 8.3 than at pH 7.4, which correlates with the above observation that the lipid-solubilisation efficiency of SMA(2:1) under equilibrium conditions is enhanced with increasing pH (Fig. 5F), although solubilisation is slower under such alkaline conditions^15^. On a broader note, this highlights the usefulness of *in vitro* lipid-bilayer studies, particularly those performed under equilibrium rather than kinetically controlled conditions, for tuning the solubilisation of more complex, biological membranes containing considerable amounts of proteins. In spite of the overall high protein-extraction yields enabled by all three copolymers, it is also obvious that different copolymers solubilise various proteins to different extents (Fig. 6). In particular, DIBMA tends to preferentially extract larger proteins rather than smaller ones, which could be owed to the fact that this copolymer forms larger nanodiscs than the two SMA variants^18^. Finally, it should be noted that these protein-extraction trials were performed at polymer concentrations of 2.5% (*w*/*v*), which corresponds to the “default” concentration typically used in the literature^14^ but is far beyond the SOL boundaries determined using model lipid membranes (cf. Figure 2). Thus, it is conceivable that lower polymer concentrations could be employed for membrane-protein extraction without compromising yield.

## Summary and Conclusions

Amphiphilic copolymers that can solubilise proteins and lipids into nanoscale bilayer environments have recently opened new avenues in membrane research. While various types of SMA have been used over the past few years, there is a clear trend in the field to focus on SMA(2:1)^14^. This motivated us to undertake a systematic characterisation of the equilibrium membrane-solubilisation behaviour of SMA(2:1) based on approaches previously applied to SMA(3:1)^16, 17^ and DIBMA^18^. Herein, we showed that

- SMA(2:1) is an efficient solubiliser of lipid membranes, as indicated by low saturating and solubilising polymer/lipid ratios and the corresponding vesicle-to-nanodisc transfer Gibbs free energies;
- experimental conditions are important determinants of solubilisation behaviour, as SMA(2:1) is, from a thermodynamic viewpoint, more efficient at pH 8.3 than at near-neutral pH values, even though the solubilisation process is slower at elevated pH;
- SMA(2:1) represents a milder membrane solubiliser than the more hydrophobic SMA(3:1) variant but is harsher than the more hydrophilic, aliphatic copolymer DIBMA, as gauged from their effects on lipid thermotropic phase behaviour;
- the total amounts of membrane proteins extracted from native *E. coli* membranes are highest for SMA(2:1), although some proteins may be solubilised more efficiently or more mildly by DIBMA or, possibly, other copolymers.

## Electronic supplementary material

Supplementary Information
