# Supplementary material for: Thermodynamics of nanodisc formation mediated by styrene/maleic acid (2:1) copolymer
Source: Sci Rep. 2017 Sep 14;7:11517. doi: 10.1038/s41598-017-11616-z (PMC5599547; doi:10.1038/s41598-017-11616-z)
Supplement: Supplementary file 1 — Supplementary Information [file 41598_2017_11616_MOESM1_ESM.pdf]

Supplementary Information

Thermodynamics of nanodisc formation mediated by styrene/maleic acid (2:1) copolymer

Anne Grethen[1], Abraham Olusegun Oluwole[1,2], Bartholomäus Danielczak[1], Carolyn Vargas[1] & Sandro Keller[1,*]

[1] Molecular Biophysics, University of Kaiserslautern, 67663 Kaiserslautern, Germany

[2] Department of Chemistry, University of Ibadan, 200284 Ibadan, Nigeria

*mail@sandrokeller.com

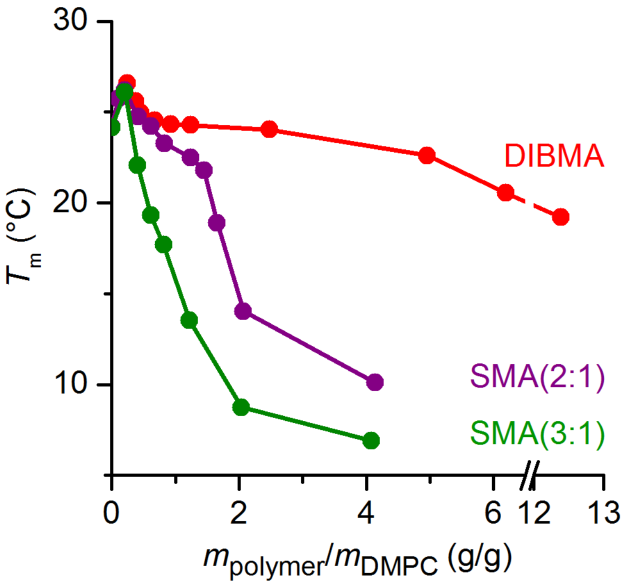

Supplementary Figure 1. Gel-to-fluid phase transition temperatures, T_m, as functions of polymer/DMPC *mass* ratios. The same T_m values as functions of polymer/DMPC *molar* ratios are shown in Fig. 4B in the main text.

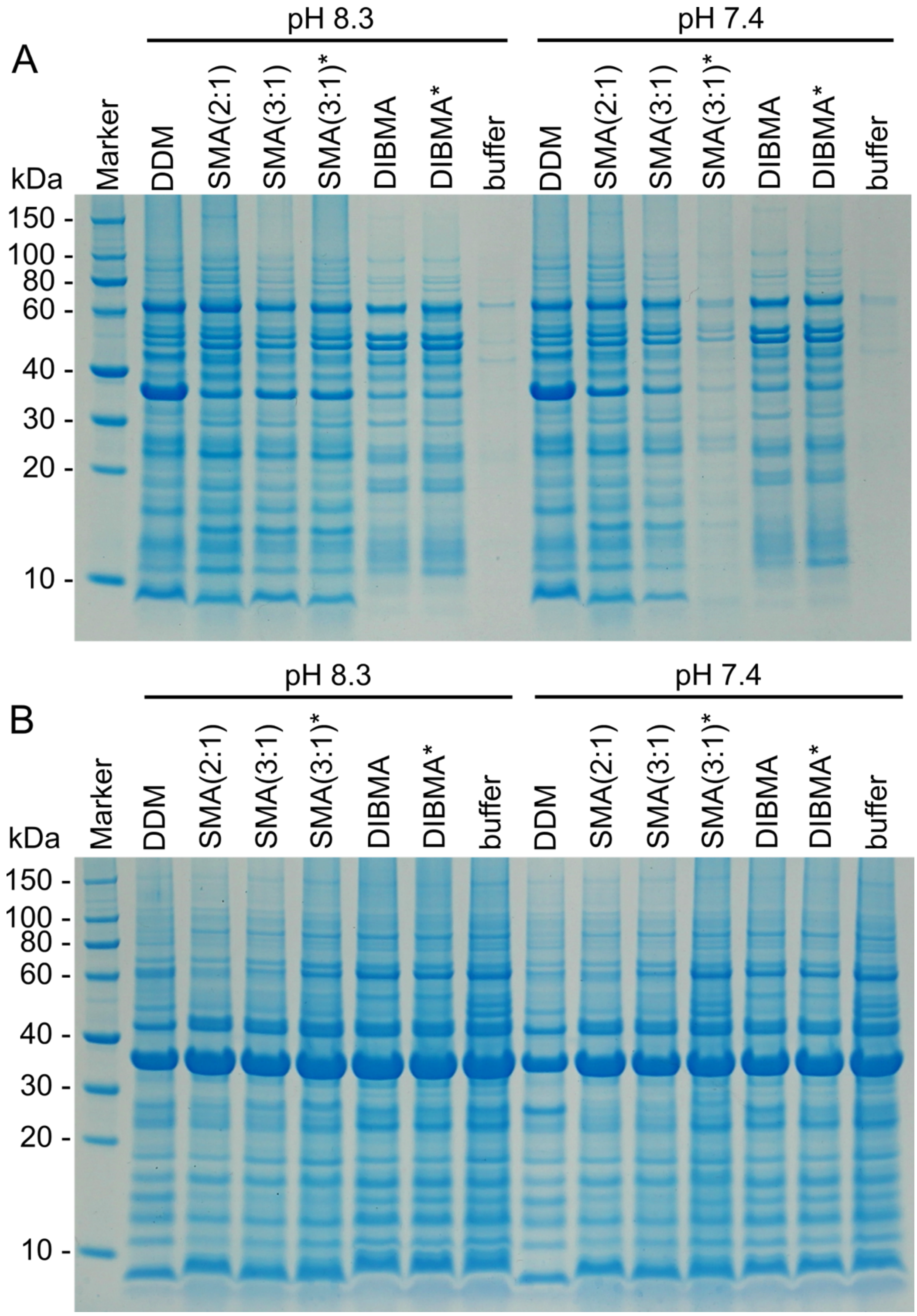

Supplementary Figure 2. Full-length SDS-PAGE showing the solubilisation of *E. coli* BL21(DE3) membranes by 10 mM (0.5% (*w*/*v*)) DDM, 9.3 mM (2.5% (*w*/*v*)) SMA(2:1), 6.3 mM (2.5% (*w*/*v*)) SMA(3:1), or 3.0 mM (2.5% (*w*/*v*)) DIBMA. (A) Solubilised membrane-protein fractions (i.e., supernatants) after removal of unsolubilised material by ultracentrifugation. Data for DDM, SMA(3:1), and DIBMA are reproduced from Oluwole et al. *Angew. Chem. Int. Ed.* **56**, 1919–1924 (2017). A cropped version of this gel is shown in Figure 6 of the main text. (B) Unsolubilised membrane fragments (i.e., resuspended pellets) after ultracentrifugation for 1 h at 100,000 *g*. Note that relative solubilisation yields are generally low under these conditions but increase with decreasing amounts of *E. coli* membranes used for extraction. *Solubilisation trials performed at an elevated ionic strength of 500 mM NaCl as compared with standard buffer conditions (i.e., 50 mM Tris, 200 mM NaCl, 20°C). Reduced protein-extraction yields were most likely due to aggregation of SMA(3:1) at 500 mM NaCl (Scheidelaar et al. *Biophys. J.* **111,** 1974–1986 (2016).
